# Highly conserved type 1 pili promote enterotoxigenic *E*. *coli* pathogen-host interactions

**DOI:** 10.1371/journal.pntd.0005586

**Published:** 2017-05-22

**Authors:** Alaullah Sheikh, Rasheduzzaman Rashu, Yasmin Ara Begum, F. Matthew Kuhlman, Matthew A. Ciorba, Scott J. Hultgren, Firdausi Qadri, James M. Fleckenstein

**Affiliations:** 1 Molecular Microbiology and Microbial Pathogenesis Program, Division of Biology and Biomedical Sciences, Washington University School of Medicine, Saint Louis, Missouri, United States of America; 2 Infectious Diseases Division, International Centre for Diarrhoeal Disease Research, Bangladesh (icddrb), Mohakhali, Dhaka, Bangladesh; 3 Division of Infectious Disease, Department of Internal Medicine, Washington University School of Medicine, Saint Louis, Missouri, United States of America; 4 Division of Gastroenterology, Department of Internal Medicine, Washington University School of Medicine, Saint Louis, Missouri, United States of America; 5 Department of Molecular Microbiology, Washington University in Saint Louis, Saint Louis, Missouri, United States of America; 6 Center for Women’s Infectious Disease Research (CWIDR), Washington University in Saint Louis, Saint Louis, Missouri, United States of America; 7 Medicine Service, Veterans Affairs Medical Center, Saint Louis, Missouri, United States of America; Beijing Institute of Microbiology and Epidemiology, CHINA

## Abstract

Enterotoxigenic *Escherichia coli* (ETEC), defined by their elaboration of heat-labile (LT) and/or heat-stable (ST) enterotoxins, are a common cause of diarrheal illness in developing countries. Efficient delivery of these toxins requires ETEC to engage target host enterocytes. This engagement is accomplished using a variety of pathovar-specific and conserved *E*. *coli* adhesin molecules as well as plasmid encoded colonization factors. Some of these adhesins undergo significant transcriptional modulation as ETEC encounter intestinal epithelia, perhaps suggesting that they cooperatively facilitate interaction with the host. Among genes significantly upregulated on cell contact are those encoding type 1 pili. We therefore investigated the role played by these pili in facilitating ETEC adhesion, and toxin delivery to model intestinal epithelia. We demonstrate that type 1 pili, encoded in the *E*. *coli* core genome, play an essential role in ETEC virulence, acting in concert with plasmid-encoded pathovar specific colonization factor (CF) fimbriae to promote optimal bacterial adhesion to cultured intestinal epithelium (CIE) and to epithelial monolayers differentiated from human small intestinal stem cells. Type 1 pili are tipped with the FimH adhesin which recognizes mannose with stereochemical specificity. Thus, enhanced production of highly mannosylated proteins on intestinal epithelia promoted FimH-mediated ETEC adhesion, while conversely, interruption of FimH lectin-epithelial interactions with soluble mannose, anti-FimH antibodies or mutagenesis of *fimH* effectively blocked ETEC adhesion. Moreover, *fimH* mutants were significantly impaired in delivery of both heat-stable and heat-labile toxins to the target epithelial cells *in vitro*, *and* these mutants were substantially less virulent in rabbit ileal loop assays, a classical model of ETEC pathogenesis. Collectively, our data suggest that these highly conserved pili play an essential role in virulence of these diverse pathogens.

## Author summary

Enterotoxigenic *Escherichia coli* (ETEC) infections contribute substantially to death and morbidity due to diarrheal illness and are associated with serious sequelae including malnutrition, stunted growth, and intellectual impairment among young children in developing countries. Effective engagement of intestinal epithelial cells is essential for ETEC pathogenicity. Consequently, pathovar specific plasmid-encoded adhesin structures known as colonization factors (CFs) have been a principal target for vaccines. However, tremendous inter-strain variation in the carriage of gene clusters encoding different CFs and significant antigenic diversity of the CF adhesins has posed a challenge to vaccine development. In contrast, type 1 pili are encoded by the *fim* operon located in the chromosome of most ETEC strains and are highly conserved. While type 1 pili are known to play a critical role in virulence of extraintestinal pathogenic *E*. *coli*, the present studies represent the first detailed examination of the contribution of these pili in ETEC pathogenesis. Here we demonstrate that ETEC type 1 pili are essential for optimal interactions with intestinal epithelia and that they play a critical role in virulence. These data may inform additional approaches toward development of broadly protective vaccines for these pathogens of global importance.

## Introduction

Among young children under five years of age in developing countries, diarrhea is a leading cause of morbidity and mortality. Enterotoxigenic *E*. *coli* (ETEC) is one of the most common causes of moderate to severe diarrheal illness and deaths due to diarrhea in young children and incidentally is also the leading bacterial cause of diarrhea [1]. These bacteria are also a leading cause of hospitalization due to severe diarrhea in adults in developing countries [2] and are perennially the predominant cause of diarrheal illness among travelers to the endemic regions [3, 4]. Additionally, ETEC infections contribute substantially to the burden of diarrheal illness associated with sequelae of malnutrition [5, 6], stunted growth [7] and impaired cognitive development [8]. The effects of ETEC infections also appear to be more critical in malnourished children [5]. Thus these pathogens contribute to a complex pattern of poverty, repeated enteric infections, environmental enteropathy [9], and developmental impairment.

ETEC are defined by the production of heat-labile (LT) and/or heat-stable (ST) enterotoxins [10], and virulence requires successful delivery of these toxins to cognate receptors on target intestinal epithelial cells. LT binds to cell surface GM1 gangliosides, and following cellular entry this toxin activates production of host cAMP; while ST peptides bind guanylate cyclase C, stimulating production of cGMP [11]. Resulting increases in intracellular concentrations of these cyclic nucleotides modulate ion channels on the surface of intestinal cells leading to net losses of sodium chloride and water into the intestinal lumen and ensuing acute watery diarrhea [11, 12]. In the classical paradigm of ETEC pathogenesis, these bacteria utilize pathovar-specific fimbrial or non-fimbrial adhesins, known as colonization factors or CFs [13] which allow them to adhere and colonize the small intestine where toxin delivery occurs. However, emerging evidence would suggest that this paradigm is perhaps overly simplistic, and that there are several potential adhesins which effectively act in concert to promote ETEC engagement of the host [14].

*E*. *coli* encode a multitude of pili assembled by the **c**haperone/**u**sher **p**athway, termed CUP pili, which are important in virulence. CUP pili are tipped with specialized adhesins that recognize specific receptors with stereochemical specificity. CUP adhesins can determine both tissue tropism and the course of disease. For example, type 1 pili are encoded by the *fim* operon, and chomosomally encoded as part of the core *E*. *coli* genome [15, 16]. Type 1 pili are composite fibers comprised of a pilus rod, made up of FimA subunits arranged in a right handed helical cylinder [17]. The pilus rod is joined to a fibrillum structure tipped with the FimH adhesin that binds mannose with stereochemical specificity [16, 18–20]. FimH is critical for virulence in extraintestinal *E*. *coli*, as it has been well-established that FimH mediated adhesion enables uropathogenic *E*. *coli* (UPEC) colonization and invasion into bladder epithelial cells [21–23], as well as the formation of intracellular bacterial communities [24]. ETEC also encode pathovar-specific CUP pili (fimbriae) like CFA/I [25] that are encoded on virulence plasmids [26]. More than four decades ago, these ETEC-specific colonization factors were shown to contribute to development of diarrheal illness in humans [27], and consequently they have been the subject of intensive investigation and a major focus of ETEC vaccine development. Conversely, although early studies described possible type 1 pili expression by ETEC [28, 29], relatively little is known about the contribution of these highly conserved structures to virulence. Our more recent observation that the expression of the *fim* operon is enhanced by pathogen-host cell contact [14], prompted a thorough investigation of the potential role of type 1 pili in ETEC pathogenesis reported here.

## Results

### Type 1 pili promote ETEC binding to intestinal epithelia

**Fig 1:**
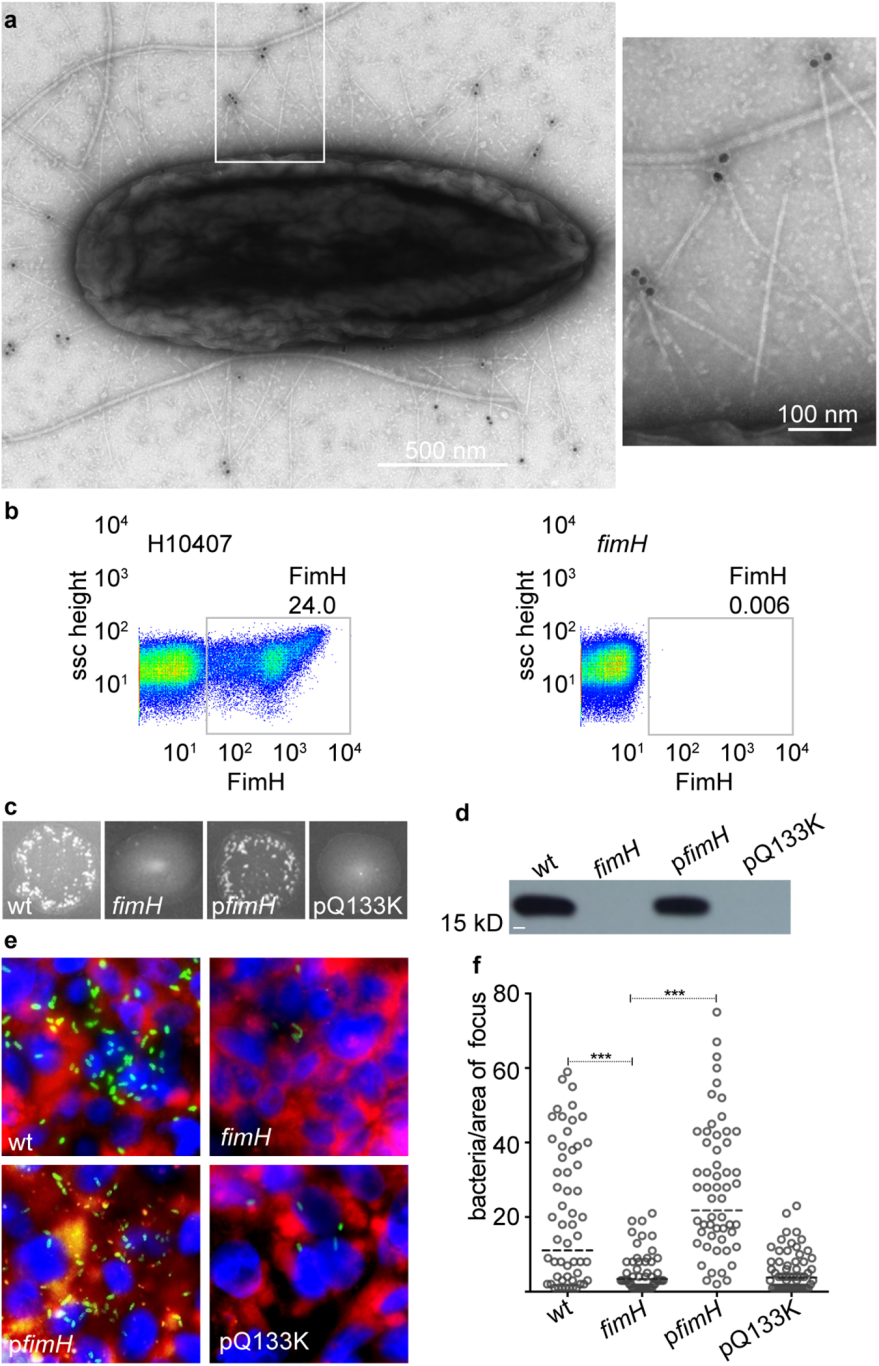
Type 1 pili expression promotes optimal adhesion of ETEC to intestinal epithelia. **a.** Transmission electron micrograph of ETEC H10407 expressing type 1 pili. The FimH tip adhesin was detected using α-FimH antibody and gold secondary antibody conjugate. **b**. Flow cytometric analysis of type 1 pili expression by ETEC H10407 and *fimH* mutants. **c.** Assessment of type 1 pili function using yeast agglutination assays. Negative yeast agglutination reflected the loss of type 1 pili activity. **d.** FimA immunoblot of type 1 pili extracts from static culture of WT ETEC, *fimH* mutants and mutants complemented with wild type *fimH* gene (p*fimH*). The *fimH* mutant complemented with a plasmid encoding a Q133K substitution in FimH is included as a negative control. **e.** Confocal microscopic images showing adhesion of WT ETEC, *fimH* mutants or complemented mutants to polarized cultured intestinal epithelia. Bacteria (anti-O78, green), cell membrane (CellMask, red), nuclei (DAPI, blue). **f.** Quantitative analysis was done by counting number of bacteria per focus area. Horizontal dashed lines represent geometric means of 3 combined individual experiments. P values were calculated by nonparametric Mann-Whitney test. *** indicates p<0.0001.

Although a number of earlier studies of ETEC suggested that these pathogens make type 1 pili (also previously referred to as type 1 somatic pili or type 1 fimbriae) [29, 30], to date there has been no systematic examination of their involvement in ETEC virulence. We therefore first performed studies to confirm the production of type 1 pili by the prototypical ETEC H10407 strain. Type 1 pili tipped with the FimH adhesin were identified on the surface of strain H10407 by transmission electron microscopy after immunogold labeling using anti-FimH antibodies (Fig 1A). Similarly, using flow cytometry we verified production of type 1 pili in H10407, but not in the corresponding *fimH* mutant strain (Fig 1B). It has previously been shown that FimH is required to initiate the assembly of type 1 pili and thus *fimH* mutants are nonpiliated [31]. The expression of type 1 pili is under the transcriptional control of an invertible promoter element that governs phase OFF and phase ON populations [32, 33]. Interestingly, mutations that inactivate FimH such as the Q133K mutation, bias the *fim* promoter towards the phase OFF state [34]. Thus, compared to the wild type H10407 strain, or p*fimH* complemented mutants, isogenic *fimH* mutants or those complemented with pQ133K, which encodes FimH with a mutation in the mannose binding site [35], were nonpiliated and incapable of yeast agglutination, a phenotypic assay for expression of type 1 pili [36] (Fig 1C) and only the wild type and *fimH* complemented mutants exhibited demonstrable type 1 pili expression detected by anti-type 1 pili antibodies in immunoblots (Fig 1D). Next, using polarized cultured intestinal epithelia (CIE) derived from the C2BBe1 clone of Caco-2 cells which produce apical brush borders with defined microvilli similar to human intestinal enterocytes [37], we demonstrated that production of FimH was required for effective adhesion. Mutants lacking *fimH* were significantly less adherent than wild type ETEC (p<0.0001) (Fig 1E and 1F), while episomal expression of the *fimH* gene (p*fimH*), but not the mutant *fimH* allele (p*Q133K*), restored adhesion. Additionally, H10407-*fimH*:Q133K, which contains the Q133K mutant allele of *fimH* in the chromosome, abrogated yeast agglutination activity and demonstrated significant decrease in adhesion to intestinal cells (S1A Fig). Similarly, mutants lacking the *fimA* gene encoding the major type 1 pili pilin subunit exhibited loss of functional type 1 pili in yeast agglutination assays, resulting in significant reduction of adhesion to the CIE (S1B Fig).

### Inhibition of type 1 pili mediated interactions reduces ETEC adhesion

**Fig 2:**
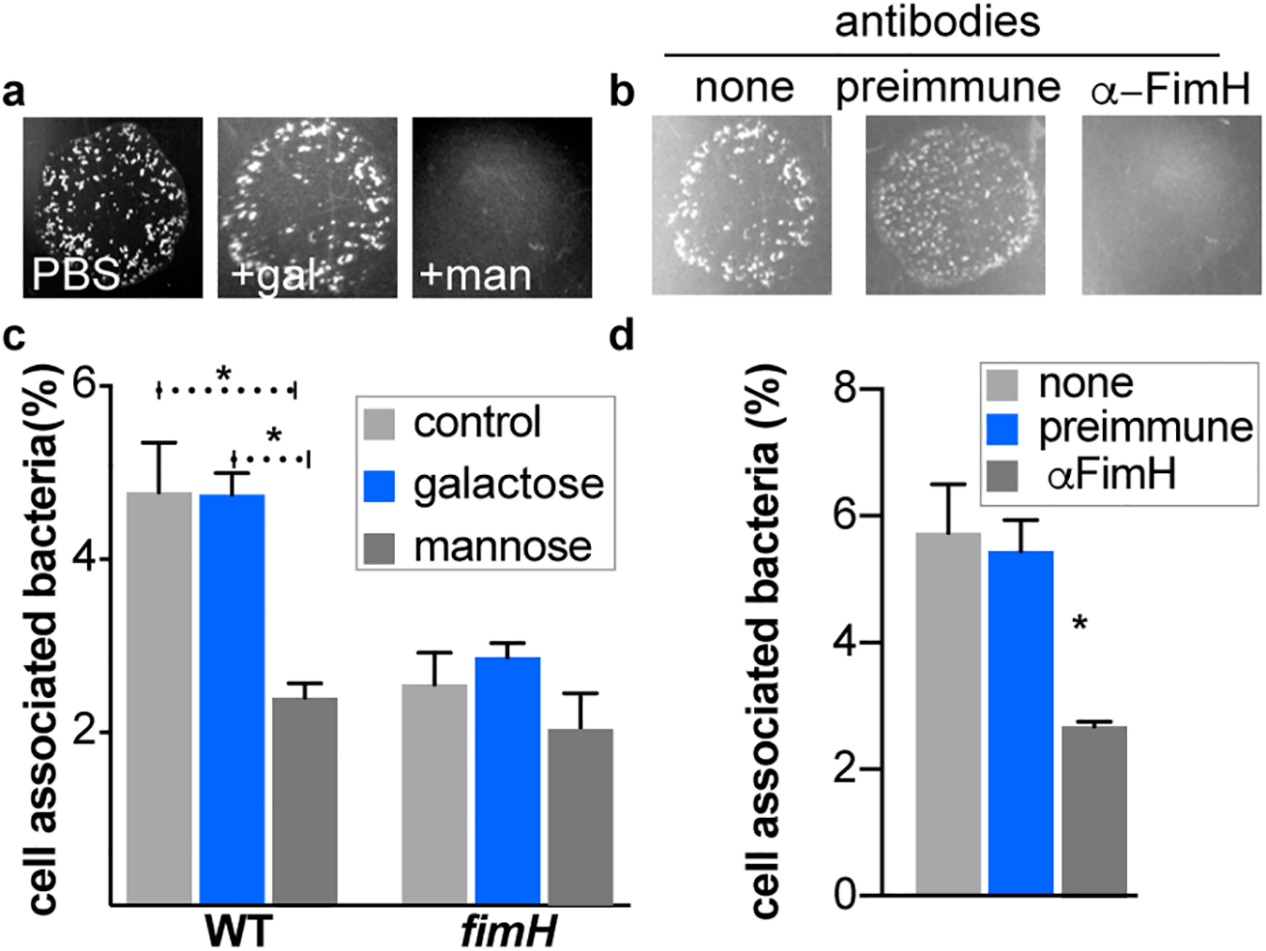
Inhibition of type 1 pili mediated interaction impairs ETEC adhesion. Yeast agglutination assay of WT ETEC (**a)** is inhibited by methyl-α-D-mannose (man) but not equimolar concentrations of methyl-α-D-galactose (gal) control sugar. **b.** Anti-FimH antibodies, generated against the lectin domain of FimH, but not pre-immune sera inhibit yeast agglutination. **c.** *In vitro* adhesion of WT ETEC or *fimH* mutants in the absence or presence of methyl-α-D-galactose or methyl-α-D-mannose. **d**. WT ETEC adhesion is inhibited by anti-FimH antibodies. The percentage of cell associated bacteria represents the proportion of bacteria associated with the CIE at the end of 1 h relative to the inoculum. Bars represent mean values + SEM (n = 5). P values were calculated by nonparametric Mann-Whitney test. * indicates p<0.05.

We found that methyl-α-D-mannose but not the methyl-α-D-galactose control sugar, inhibited FimH mediated yeast agglutination by ETEC (Fig 2A) and ETEC adhesion to epithelial cells (Fig 2C). Similarly, antibodies generated against the lectin domain of FimH (α-FimH), but not control antibodies, separated from pre-immune sera, inhibited FimH mediated yeast agglutination with wild type bacteria expressing type 1 pili (Fig 2B) and significantly inhibited ETEC adhesion to intestinal cells (Fig 2D). Collectively, these data support the idea that ETEC utilize type 1 pili to engage intestinal epithelia.

### ETEC FimH adhesin binds to intestinal epithelia

**Fig 3:**
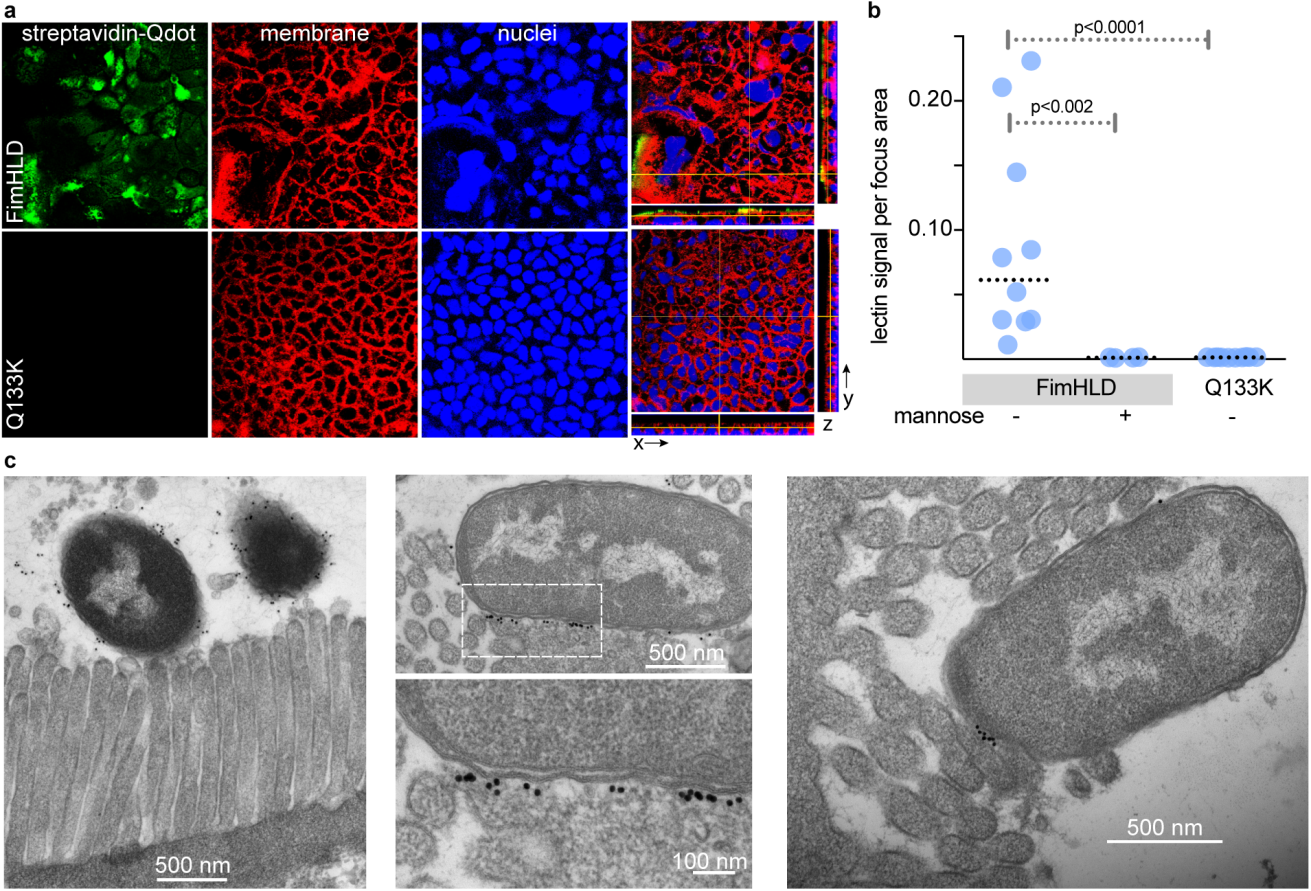
FimH adhesin of ETEC interacts with intestinal epithelial cells. **a.** Confocal microscopy images show binding of the biotinylated FimH lectin domain (FimHLD) or FimHLD:Q133K to the apical surface cultured intestinal epithelium (CIE). Biotinylated FimHLD was detected with streptavidin-conjugated fluorescent nanocrystals (Qdot, green); plasma membranes were stained with CellMask (red) and nuclei with DAPI (blue). Image at right shows three dimensional reconstruction of z stacks of CIE following interaction with FimHLD or the mutant protein. **b.** Quantitative analysis of FimHLD binding to CIE represented in panels **a** using Volocity three-dimensional (3D) image analysis software (version 6.2; PerkinElmer, Inc.). P value was calculated using nonparametric Mann-Whitney testing. **c.** Immunoelectron microscopy images of CIE infected with ETEC H10407. Left panel, microvilli structure at the apical surface of the CIE; right panels show immunogold labeling of FimH localized to the ETEC-host interacting surface.

We next examined the ability of the FimH tip adhesin to directly engage the intestinal epithelial surface. The purified lectin domain of FimH (FimHLD, 17 kD), representing amino acid residues 1–154 of mature FimH, bound to the apical surface of the CIE. In contrast, binding of FimHLD:Q133K, which lacks mannose binding activity, was markedly diminished (Fig 3A and 3B) as was binding of the wild type FimHLD protein in the presence of exogenous mannose (Fig 3B).

ETEC are noninvasive luminal pathogens thought to engage the microvilli at the apical surface of intestinal epithelial cells. Presumably, the FimH lectin can promote this engagement by interacting with mannosylated glycoconjugates on the glycocalyx covering the microvilli. Indeed, using transmission electron microscopy, we identified FimH by immunogold labeling at the ETEC-microvillus interface (Fig 3C).

### Surface mannosylation enhances FimH-mediated ETEC-epithelial interaction

**Fig 4:**
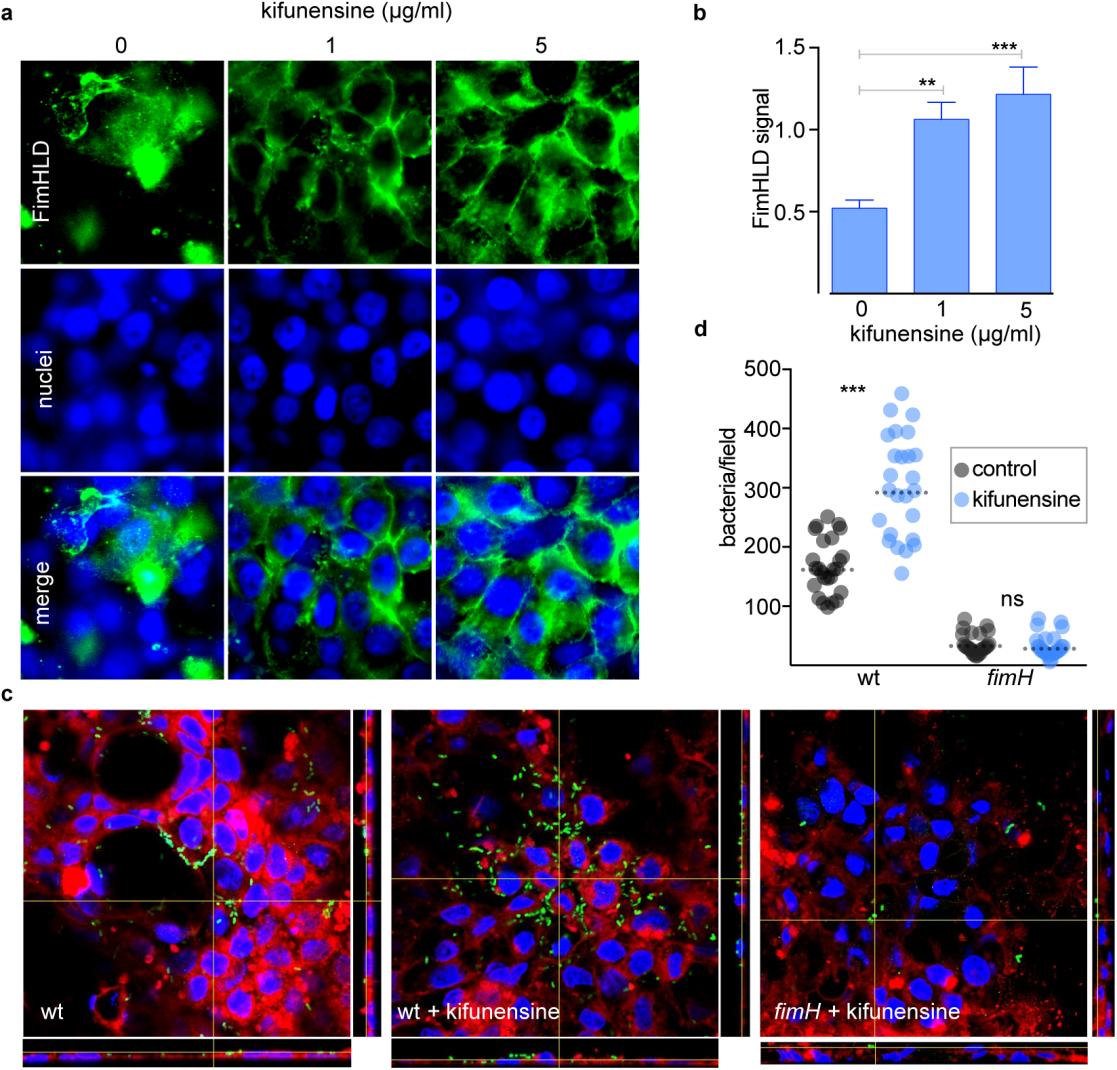
Enhanced presentation of mannosylated glycoproteins increases FimH binding and ETEC adhesion. **a.** Confocal microscopy images detecting FimHLD binding to the kifunensine treated CIE. Biotinylated FimHLD was detected with streptavidin-conjugated fluorescent nanocrystals (Qdot, green) and nuclei with DAPI (blue). **b.** Quantitative analysis of FimHLD binding to CIE represented in panels **a** using Volocity three-dimensional (3D) image analysis software (version 6.2; PerkinElmer, Inc.). Data represent mean ± standard deviation of results of 3 independent experiments each with triplicate wells per concentration tested (n = 9). **c.** Confocal microscopic images showing ETEC adhesion to kifunensine treated CIE. The CIE grown on trans-well filters were treated with kifunensine and infected with WT ETEC or *fimH* mutants. One hour post infection wells were processed for microscopic examination. Bacteria (green), cell membrane (red), DAPI (blue). **d.** Quantitative analysis was done by counting number of bacteria present per focus area. Horizontal dashed lines represent geometric mean of 25 total data points combined from 2 replicate experiments. P values were calculated by nonparametric Mann-Whitney test. *** indicates p<0.0001.

Because FimH interacts with mannosylated receptors on the epithelial surface, we examined the impact of enhanced glycoprotein mannosylation on ETEC pathogen host interactions. Following CIE treatment with kifunensine, an α-mannosidase class 1 enzyme inhibitor which enhances the display of high-mannose glycoproteins [38], we observed a significant increase in FimHLD binding (Fig 4A and 4B). Likewise, WT ETEC adhesion to the kifunensine treated CIE was enhanced relative to the untreated control CIE (p<0.0001) (Fig 4C and 4D). However, kifunensine treatment had no impact on adhesion of the *fimH* mutant. These data further suggested that availability of mannosylated glycoproteins on the host cell surface promotes ETEC adhesion through FimH.

### Type 1 pili act in concert with CFA/I fimbriae for optimal adhesion

Because ETEC H10407 expresses both type 1 pili and plasmid encoded CFA/I fimbriae specific to the ETEC pathovar, we investigated whether these CUP structures acted cooperatively in facilitating adhesion to intestinal epithelia. The CFA/I fimbriae are encoded by the *cfaABCE* operon in which *cfaE* encodes the CfaE tip adhesin [26, 39, 40]. Interestingly, mutations in either *fimH* or *cfaE* significantly reduced adherence of ETEC compared to wild type H10407, suggesting that both pili participate in ETEC adhesion (S2 Fig). Mutants lacking both the type 1 pili and the CFA/I tip adhesin genes (*fimH-cfaE*) demonstrated further reduction in adhesion compared to either of the single mutants (S2 Fig). Although additional data are needed to define the precise respective contributions of the chromosomally encoded type 1 pili and the plasmid encoded pathovar specific CFA/I, these initial data support our hypothesis that the structures act in concert to facilitate adhesion.

### Type 1 pili mediate adhesion to human small intestinal enteroids

**Fig 5:**
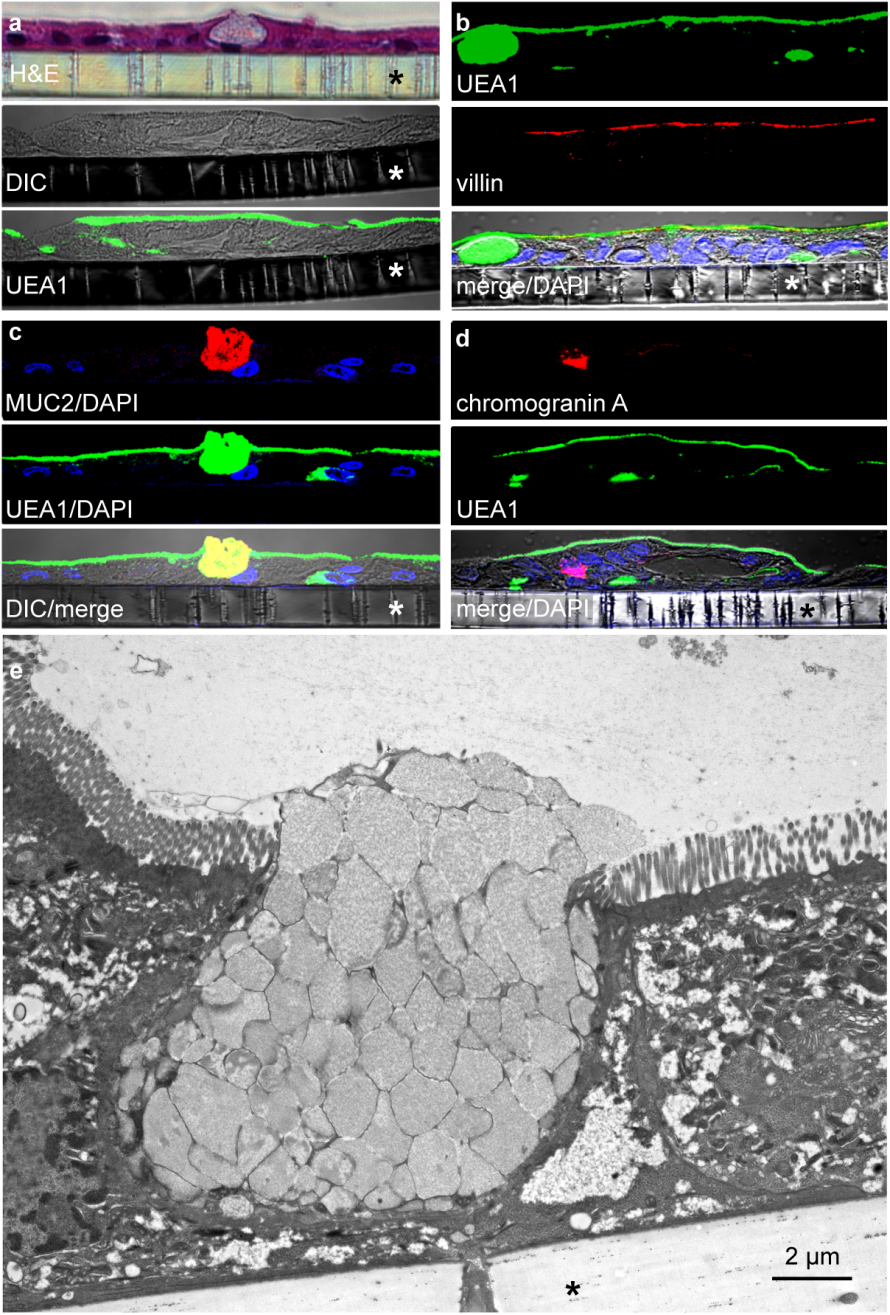
Properties of human enteroid-derived small intestinal monolayers. **a.** Hematoxylin and eosin staining, DIC, and UEA1 lectin immunofluorescence confocal microscopic images showing formation of continuous monolayers. The apical surface is detected in the bottom image with FITC conjugated UEA1 lectin. **b.** UEA1 and anti-villin 1 antibody immunofluorescence in Laser Scanning Confocal Microscopy (LSCM) images from sections of polarized small intestinal enteroid monolayers. Nuclei (DAPI) are shown in blue. **c.** LSCM of sections showing MUC2 immunofluorescence (red) in probable goblet cell and co-localization with UEA1 in the merged image. **d.** Chromogranin A positive cells (red). **e.** Transmission electron microscopy (3000x) of polarized small intestinal enteroid monolayer sections showing a goblet cell flanked by enterocytes with distinct microvilli on the apical surface. Transwell filters in sections are indicated by *.

While the CIE used in this study possess some features of normal intestinal epithelial cells, they are derived from metastatic colon cancer cells which fail to represent the diversity of cell types present in intestinal epithelium. Enteroids, derived from human intestinal stem cells collected from healthy volunteers [41–43], can recapitulate many aspects of normal physiology and preserve features of human intestinal epithelium. These include presentation of different cell types including enterocytes, goblet cells, Paneth cells, and endocrine cells [44–46]. Therefore, to further assess the contribution of type 1 pili in ETEC adhesion to intestinal epithelia we established polarized intestinal epithelial monolayers derived from ileal specimens obtained from normal adult human subjects. In these enteroid-derived monolayers we were able to identify enterocytes with a defined brush border and distinct microvilli on the apical surface as well as goblet cells, and chromogranin A positive cells suggesting that they faithfully reproduce many features of surfaces normally presented to bacteria within the intestine (Fig 5A–5E).

**Fig 6:**
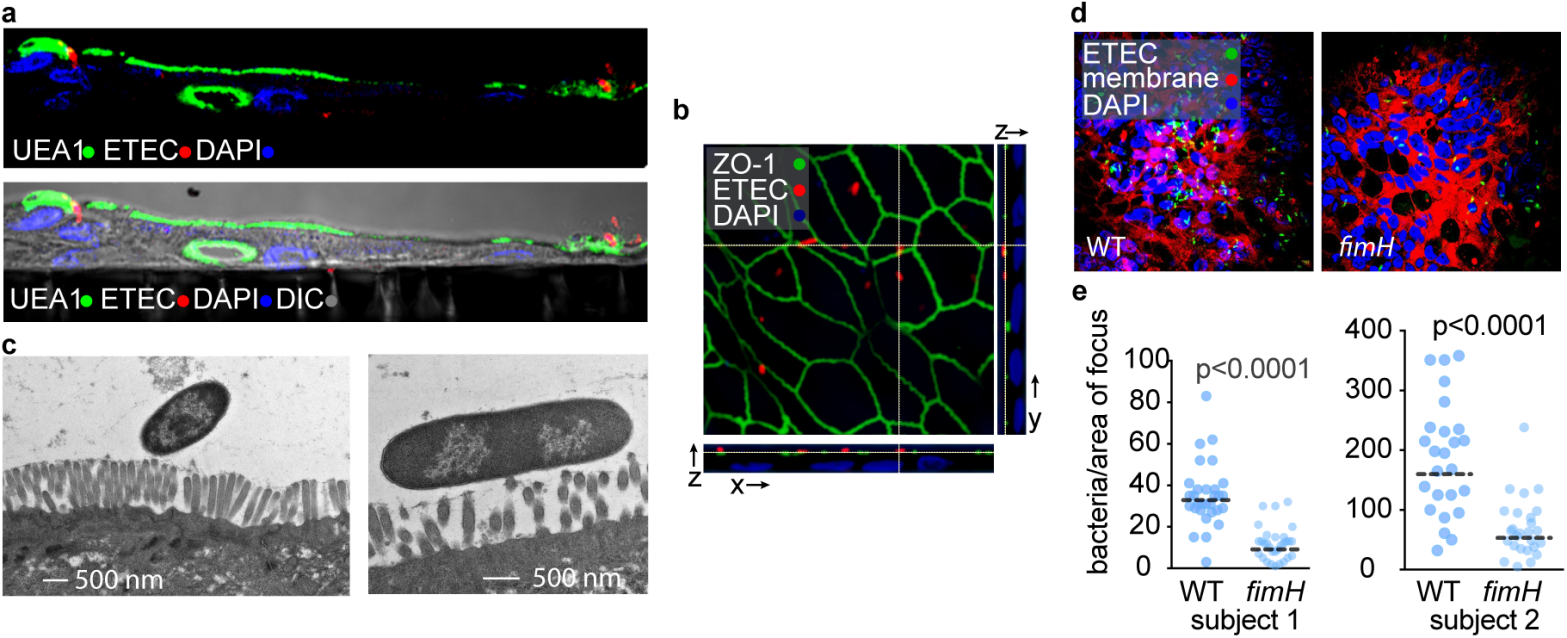
Type 1 pili are required for optimal adhesion to small intestinal epithelia. **a.** Representative laser scanning confocal microscopy (LSCM) images of sections prepared from human small intestinal enteroid-derived polarized monolayers infected with WT H10407 (red, anti-O78). Surface staining with the UEA1 lectin is shown in green. Nuclei are stained in blue (DAPI). **b.** Three dimensional reconstruction of LSCM z stacks of ETEC H10407 infected polarized monolayers showing the distribution of zonula occludens-1 (ZO-1, green) at the apical surface. Bacteria were visualized with anti-O78 (red) and nuclei are stained with DAPI (blue). **c.** Transmission electron microscopy images of ETEC H10407 adhering to microvilli on the surface of small intestinal monolayers (magnification 7500x and 15000x for left and right images respectively). **d.** LCSM images of wild type (wt) versus *fimH* mutant bacteria adherent to the surface of small intestinal enteroids Bacteria were visualized with anti-O78 (green) and cell membranes with CellMask (red), nuclei (DAPI, blue). **e.** Quantitative analysis of ETEC adhesion to enteroid-derived monolayers represented in panel **d**. Each dot plot represents adhesion data obtained using ileal cell derived from two individual subjects. Horizontal lines represent geometric means of data combined from 2 independent experiments. P values were calculated by nonparametric Mann-Whitney testing.

Similar to our studies of CIE, we found that wild type ETEC adhered to the surface of stem cell derived polarized small intestinal (ileal) monolayers (Fig 6A and 6B) in close approximation with the microvillus surface (Fig 6C), and that mutation of *fimH* significantly attenuated adherence relative to the wild type parent strain (Fig 6D). Wild type bacteria adhered well to polarized monolayers of enteroid collected from multiple individuals, while the *fimH* mutant was persistently deficient in its ability to adhere to all target epithelia relative to the parent strain (Fig 6E). These studies suggested that type 1 pili of ETEC potentially play an important role in specifically directing bacterial interaction with the small intestine where release of toxins is thought to provoke the efflux of water and salt that lead to diarrhea.

### Type 1 pili promotes optimal toxin delivery to intestinal cells *in vitro*

**Fig 7:**
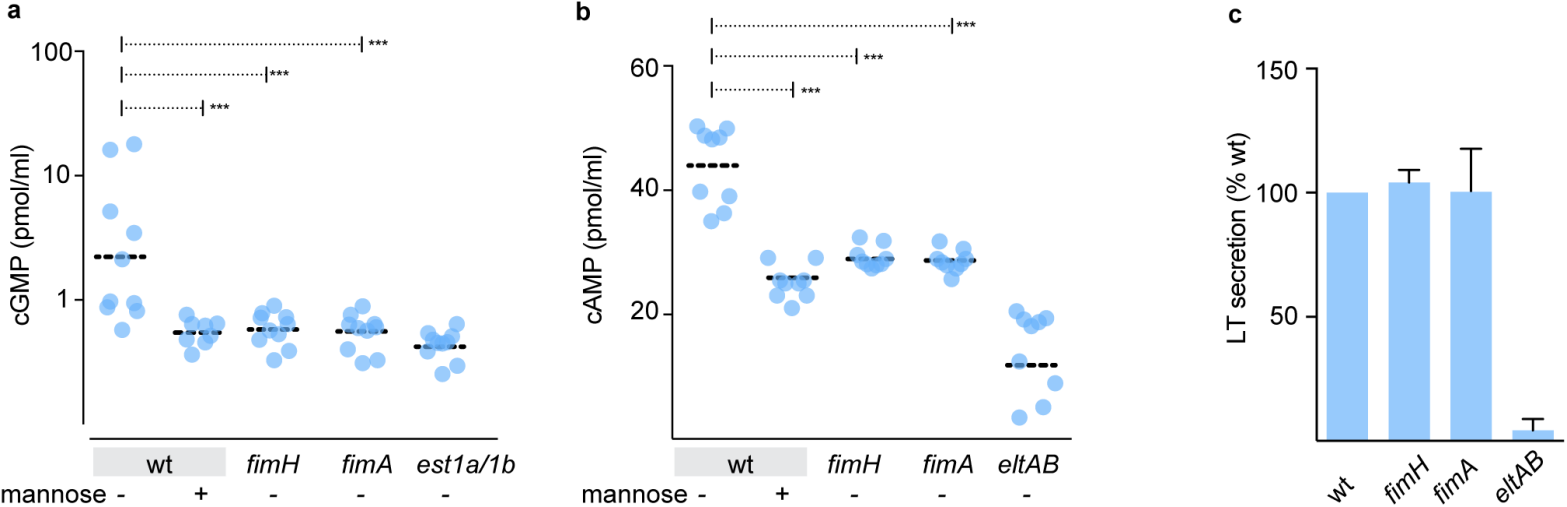
Type 1 pili mediated interactions enhance toxin delivery. **a.** Quantification of intracellular cGMP in infected cells. Cells were infected with WT ETEC in the absence or presence mannose sugar or with *fimH* or *fimA* mutants. Cells infected with *estH/estP* mutants which lack production of both heat stable toxins ST-H (ST-1b) and ST-P (ST-1a), represent basal level of cGMP in cells. **b.** Quantification of the amount of intracellular cAMP in infected cells. Cells were infected with WT ETEC in the absence or presence mannose sugar or with *fimH* or *fimA* mutants. Cells infected with *eltAB*, LT mutants, represent basal level of cAMP in cells. **c.** LT secretion by different mutants. Each bar represent mean with SEM (error bar) of 2 experiments consisting of 5 replicates per experiment for each strain. All P values were calculated by nonparametric Mann-Whitney test. *** p<0.0001.

Close contact of ETEC with target epithelial cells is essential for efficient delivery of its enterotoxins [47, 48]. To investigate the impact of type 1 pili mediated ETEC-host interactions on toxin delivery, we measured the intracellular production of cAMP and cGMP, cyclic nucleotide second messenger markers for delivery of LT and ST, respectively, in infected cells. cAMP and cGMP was significantly increased in target cells infected with WT H10407 relative to *fimH* or *fimA* mutants (Fig 7A and 7B). Methyl-α-D-mannose significantly reduced levels of intracellular cGMP and inhibited cAMP activation in target cells infected with WT H10407 (Fig 7A and 7B). These effects were observed despite wild type levels of heat-labile toxin being produced by the mutants (Fig 7C). Together, these data suggested that type 1 pili are required for optimal delivery of both heat-labile and heat-stable enterotoxins to the target epithelial cells.

### Type 1 pili are required for ETEC virulence

**Fig 8:**
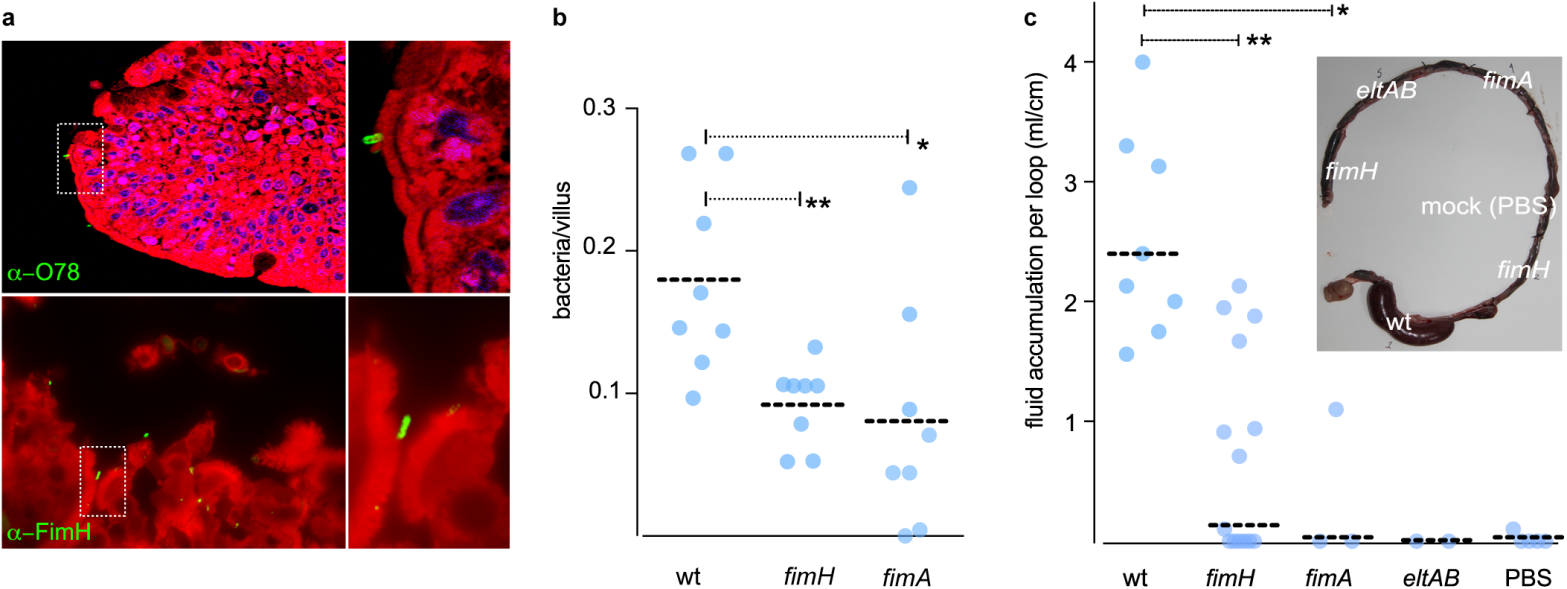
Type 1 pili are required for virulence in the rabbit ileal loop assay. Type 1 pili are required for optimal bacterial engagement of rabbit intestinal epithelia. **a.** sections of rabbit ileum in which attached bacteria (green) are identified with anti-O78 (top panel) or anti-FimH (bottom panel). Nuclei are stained with DAPI (blue) and membranes are stained with CellMask (red). **b.** bacteria adherent to the ileal mucosal surface following infection with wild type ETEC H10407 or *fimH* and *fimA* mutants. **c.** Type 1 pili are required for toxicity in the rabbit ileal loop assay. Shown in the graph is the amount of fluid accumulation in each loop infected with WT or *fimH* or *fimA* mutants 18 h post inoculation. Loops infected with *eltAB* mutants or mock infected (PBS) were used as controls. Data represent the summary of experiments from 7 different rabbits (n = 7). Inset image shows infected ileal loops from one representative experiment. Each loop in the inset image is labeled with the infecting bacterial strain or with the negative control (PBS).

We next examined the contribution of type 1 pili to pathogenesis in the rabbit ileal loop model, a classical model of virulence for *V*. *cholerae* and ETEC [49]. Both *fimH* and *fimA* mutants were significantly less adherent than the WT H10407 to rabbit ileal intestinal epithelium (Fig 8A and 8B). Likewise, while considerable fluid accumulation, a hallmark of ETEC virulence, was observed in loops infected with WT H10407 (Fig 8C) no demonstrable fluid accumulation was observed in control loops infected with *eltAB* mutants [47] which do not make LT toxin (S1 Table), or those containing only PBS, and we detected significantly less fluid accumulation in the loops infected with either the *fimH* or *fimA* mutants (Fig 8C), suggesting that type 1 pili mediated pathogen-host interactions contribute to ETEC virulence in this model.

### Type 1 pili are highly conserved in diverse clinical ETEC isolates

To investigate the prevalence of functional type 1 pili expression in clinical ETEC isolates, we tested 174 geographically and phylogenetically disparate clinical isolates including recently sequenced [50] strains using yeast agglutination assays. Overall, the majority (76%) of the clinical isolates demonstrated yeast agglutination activity indicative of preserved functional type 1 pili expression among ETEC. Importantly, we observed type 1 pili expression in isolates possessing each of the major colonization factors (CFs), and in 82% of isolates without any recognizable CFs (Table 1). Overall, these data suggest that functional type 1 pili are highly conserved in diverse ETEC clinical isolates.

**Table 1:** Relationship of functional type 1 pili expresion to colonization factors.

CF designation(s)	CF expression		Type 1 fimbrial expression^a^	
	number	%	number	%
CFA/I, CS21	28	16	26	93
CS1, CS3, CS21	15	8.6	2	13
CS7	14	8	14	100
CS2, CS3, CS21	11	6.3	10	91
CS5, CS6	9	5.2	9	100
CFA/I	7	4	7	100
CS1, CS3	6	3.4	2	33
CS21	6	3.4	2	33
CS17	5	2.9	5	100
CS20, CS21	5	2.9	5	100
CS4, CS6	5	2.9	1	20
CS2, CS3	4	2.3	2	50
CS14	3	1.7	2	67
CS6, CS21	3	1.7	3	100
CS1	2	1.1	2	100
CS1, CS3, CS20, CS21	2	1.1	0	0
CS6	2	1.1	2	100
CS6, CS20, CS21	2	1.1	2	100
CS6, CS8	2	1.1	2	100
CFA/I, CS20, CS21	1	0.6	1	100
CS1, CS21	1	0.6	0	0
CS12, CS20	1	0.6	1	100
CS12, CS20, CS21	1	0.6	1	100
CS19	1	0.6	1	100
CS2, CS3, CS20	1	0.6	1	100
CS2, CS3, CS20, CS21	1	0.6	0	0
CS5	1	0.6	1	100
CS5, CS6, CS21	1	0.6	1	100
Undetected	34	19.5	28	82
total + isolates	140^b^	80	133 ^b^	76

^a^ determined by mannose-sensitive yeast agglutination.

^b^ of n = 174 total isolates queried

Collectively data presented here provide evidence that type 1 pili play an essential role in the pathogenesis of these highly diverse pathogens. Similar to their well-established role in the pathogenesis of uropathogenic *E*. *coli* (UPEC) [51] where type 1 pili are required for interaction with bladder epithelia, these structures appear to be highly conserved in clinical isolates and appear to be critical for ETEC adhesion and effective engagement of host intestinal epithelia that are ultimately required for efficient delivery of effector molecules including the known toxins.

## Discussion

Enterotoxigenic *E*. *coli* are a remarkably diverse group of pathogens that share plasmid-encoded effector molecules, namely heat-labile toxin (LT) and/or heat-stable toxins (ST). In effect, ETEC pathogenesis can be summarized by the virulence features that collectively facilitate the delivery of these toxins [52]. Successful engagement of the complex landscape presented by the intestinal mucosae that includes a secreted mucus layer as well as the glycocalyx, glycoconjugates on the apical surface of the epithelium [53, 54], represents an essential step in ETEC virulence. Like other enteric pathogens, ETEC appear to employ a number of different adhesins (lectins) that recognize specific carbohydrate moieties on intestinal epithelia [55–58].

While most studies of ETEC adhesion, and consequently vaccine development, had previously focused on plasmid-encoded colonization factors, recent studies have suggested that bacterial adhesion, intestinal colonization, and toxin delivery ultimately represent very complex phenotypes involving the orchestrated deployment of a variety of pathovar specific plasmid encoded adhesins [14, 59, 60] as well as highly conserved chromosomally-encoded molecules [61] and other virulence factors including mucinases [62, 63]. Although a number of early studies had suggested that ETEC have the capacity to make type 1 pili [30, 64], their contribution to pathogenesis had not been comprehensively investigated.

Here, we demonstrate convincingly that most ETEC make type 1 pili, that these organisms utilize these highly conserved pili to engage model intestinal epithelia, where these interactions are critical for effective delivery of both heat-labile and heat-stable toxins. Additionally, our data demonstrate that type 1 pili act in concert with the plasmid-encoded pathovar specific colonization factors to promote optimal interaction of H10407 with host intestinal epithelial surfaces. The precise interactions between type 1 pili and the host cell surface have not been thoroughly delineated, however, the data included here support the involvement of the minor pilin tip adhesin subunit (FimH) in mannose-dependent engagement of one or more host cell receptors [65–68].

Before the advent of recombinant techniques to construct isogenic deletion mutants, earlier investigations of ETEC H10407-P, a plasmid-cured strain of H10407 which lacks the large virulence plasmid encoding CFA/I colonization factor, Knutton *et*. *al*. suggested that type 1 pili mediated ETEC adhesion to human intestinal biopsies in a fashion that was inhibited by exogenous mannose [28, 29]. However, it was suggested that these pili were mediating adhesion to the basolateral surface of enterocytes rather than the apical side [28]. The data presented here overcome many of the technological limitations inherent in these earlier studies and demonstrate convincingly that type 1 pili mediate adhesion of ETEC to apical surface of small intestinal enterocytes, where LT and ST bind to surface GM-1 and guanylate cyclase C receptors, respectively.

The present studies raise important questions regarding the nature of type 1 pilus production by ETEC including how these structures might work cooperatively with canonical colonization factor fimbriae, and whether ETEC might encode FimH variants[69, 70] that favor colonization of the small intestine. Addressing these issues could be relevant to understanding how type 1 pili might be targeted in vaccines.

While ETEC cause a tremendous burden of disease in low-middle income countries, and among travelers to these regions, at present, there is no suitable broadly protective vaccine to prevent infections caused by ETEC. This in part relates to substantial genetic and antigenic heterogeneity within the enterotoxigenic *Escherichia coli* pathovar. Most vaccines to date, in some form, have targeted the plasmid encoded colonization factor antigens. The remarkable heterogeneity of these antigens [71] and the fact that many ETEC [72, 73], more than half of isolates in some studies [74], do not make a recognizable colonization factor have prompted further investigation of these pathogens to define additional vaccine target antigens [75]. Our observation that ETEC isolates from a phylogenetically and geographically diverse collection of strains express functional type 1 pili can potentially inform alternative approaches to design of broadly protective immunogens.

In summary, the data presented here demonstrate that ETEC utilize type 1 pili for optimal engagement of the host intestinal epithelium and that these interactions facilitate toxin delivery to the target enterocytes essential for virulence. These studies provide an expanded view of ETEC molecular pathogenesis beyond the canonical paradigm envisioned more than 40 years ago, and potentially afford new avenues for the rational design of strategies to prevent the global burden of disease associated with these important pathogens.

## Methods

### Mutagenesis and cloning

Isogenic *fimH* and *fimA* mutants (S1 Table) were constructed using lambda red mediated recombination as previously described [76].To construct the *fimH* mutant primers jf101413.7 and jf101413.8 (S2 Table) were used to amplify the kanamycin resistance cassette from pKD4 plasmid with 60-bp tails corresponding to the DNA sequence immediately upstream and downstream of *fimH*. The resulting amplicon was then introduced into H10407 carrying the pKD46 helper plasmid for lambda red-mediated homologous recombination and mutants were selected on 50 μg/ml Kanamycin containing LB-agar plate, and tested for loss of *fimH* gene by PCR using primers jf120913.9 and jf120913.10. A complementation plasmid (p*fimH*) was constructed by amplifying the *fimH* gene with its native stop codon using primers jf120814.1 and jf120814.2 and cloning into pFLAG-CTC plasmid using infusion cloning kit (Clontech, Takara Bio, USA). A *fimA* complementation plasmid was constructed by cloning the fimA gene from H10407 into the *EcoR1* and *BamH1* sites of pTrc99A[77]. Site-directed mutagenesis of *pfimH* with primers jf031814.1 and jf031814.2 was used to change the CAA codon corresponding to the glutamine residue at position 133 of FimH to AAA codon corresponding to the lysine residue resulting in pQ133K (QuikChange, Stratagene, USA). Plasmids p*fimH* and p*Q133K* (S3 Table) were then introduced into the *fimH* mutants for complementation. All mutants were checked for motility, growth and secretion of known effector molecules including LT, EtpA and EatA.

To construct an expression plasmid for polyhistidine tagged FimH lectin domain (FimHLD), we amplified the N-terminal lectin region of *fimH* gene using primers jf042314.1 and jf042314.2. The amplicon was then cloned into pETDUET1 (In-Fusion, Clontech). Site directed mutagenesis was used to generate the mannose binding deficient FimHLD:Q133K as described above. The resulting plasmids, p*fimHLD* and p*fimHLD*:*Q133K*, were then introduced into BL21 (DE3) pLys strain for expression and purification of polyhistidine-tagged FimHLD and FimHLD:Q133K.

### Expression and purification of recombinant protein

Overnight cultures of BL21 (DE3) pLys containing p*fimHLD* or p*fimHLD*:*Q133K* were diluted 1:100 into 2 liters of terrific broth supplemented with 100 μg/ml ampicillin and grown for 3 h at 37°C to optical density of ~0.7 at a wavelength of 600 nm (OD600) then induced with 1 mM IPTG for 3 h at 30°C. Cells were harvested, lysed and his-tagged proteins were purified from the bacterial lysates by nickel affinity chromatography using HisTrap HP column (GE healthcare bioscience, PA, USA). His-tagged FimHLD and FimHLD:Q133K were further purified by size exclusion column chromatography using HiLoad16/600 Superdex 200 pg column (GE).

### Antibody generation and purification

Anti-FimH polyclonal rabbit antiserum was produced against the lectin domain of FimH as previously described [61]. Briefly, two New Zealand White rabbits were immunized (Rockland, USA) with recombinant polyhistidine-tagged FimHLD. Antibodies were separated from serum components using HiTrap columns prepacked with protein G Sepharose (GE). The resulting polyclonal antibodies were then pre-absorbed using lyophilized strain AAEC191-A [15], and affinity purification of antibody against FimHLD immobilized on nitrocellulose was performed as previously described [61, 78].

### Bacteria and cell culture

All experiments were carried out using prototypical ETEC strain H10407[79] or the isogenic mutants (S2 Table). Bacteria were grown at type 1 pili inducing conditions by following static incubation for 24 h at 37°C followed by subculturing at 1:100 for additional 24 h statically at 37°C [34] in LB media supplemented with or without antibiotic, as appropriate, unless otherwise stated. C2BBe1 cells (ATCC Accession Number CRL-2102), a subclone of the Caco-2 cell colonic adenocarcinoma line, was used for generation of the cultured intestinal epithelium (CIE). In order to generate polarized monolayers which form an apical brush border, with microvilli morphologically comparable to that of the human intestinal epithelium [37], we seeded ~2 x10^5^ C2BBe1 cells onto Transwell filters (0.4μM polystyrene membrane, 6.5mm diameter insert) in DMEM media supplemented with 10% FBS and 10 μg/ml human transferrin (Lonza, MD, USA) and grew at 37°C with 5% CO_2_ for three weeks for CIE (polarized monolayer culture). Media were changed every 2–3 days. Formation of microvilli was verified by transmission electron microscopy. Caco-2 cells (ATCC) were cultured in MEM media supplemented with 20% FBS. T-84 (ATCC) cells were cultured in DMEM/F12 (1:1) media supplemented with 5% FBS.

### Flow cytometry

Strains grown under type 1 pili inducing conditions were processed for flow cytometric analysis. For surface staining, bacterial pellets were washed once with PBS, fixed in 2% paraformaldehyde for 15 min and then incubated with 1%BSA in PBS for 30 min at RT. Bacteria were then incubated with primary antibody (α-FimH) at RT for 45 min. After washing with PBS, secondary antibody staining was performed at RT in the dark with species-specific antibodies conjugated with AlexaFluor 546 for an additional 45 min. Bacteria were then washed with PBS and resuspended in 100 μl of PBS for acquisition by flow cytometry (FACSCalibur, BD Biosciences). A minimum of 50,000 organism counts were acquired (CellQuest software, Becton Dickinson), and subjected to subsequent analysis (FlowJo, v7.6.3).

### Type 1 pili extract preparation and detection

Type 1 pili were extracted by following a previously described method with some modifications [80]. Briefly, bacteria grown in type 1 pili inducing conditions were harvested, re-suspended in 1 ml of 1 mM Tris-HCl (pH 8.0) and incubated at 65°C for 1 h with occasional vortexing and pelleted by centrifugation (15,000 × *g* for 5 min). The supernatant was then transferred to another tube, and an aliquot was precipitated in salt (300 mM NaCl and 100 mM MgCl_2_) by incubating overnight at 4°C, followed by centrifugation at 20,000 × *g* for 10 min at 4°C. The protein pellet was re-suspended in 1 mM Tris-HCl (pH 8.0). Fimbrial extracts were separated by sodium dodecyl sulfate (SDS)-15% polyacrylamide gel electrophoresis minigels (Bio-Rad). Proteins were either stained with Coomassie brilliant blue or transferred to nitrocellulose membranes (Bio-Rad) using a Mini Trans-Blot electrophoretic cell (Bio-Rad) for 60 min at 100 V. The membrane was blocked with 5% milk-PBS supplemented with 0.05% Tween 20 (Pierce). Incubations with primary (1:5,000) and secondary (1:5,000) antibodies were carried out for 1 h at RT. Chemiluminescent substrate (Clarity Western ECL substrate, Bio-Rad) was used for detection.

### Yeast agglutination assay

Yeast agglutination as a phenotypic test for the production of type 1 pili [36], was performed using the following conditions: strains to be tested were grown in 1 ml Luria broth at type 1 pili inducing conditions, harvested and adjusted to OD600 of 1.0 in PBS. Agglutination was performed on glass slides by mixing 20 μl bacteria with an equal volume of bakers’ yeast suspension adjusted to OD600 of 1 in PBS.

### Adhesion assays

For adhesion assays, bacteria to be tested were grown at type 1 pili inducing conditions and diluted at 1:10 in pre-warmed cell culture media prior to infection. Serial dilutions of each inoculum were plated on Luria agar for CFU count. Infected CIE were incubated at 37°C and 5% CO_2_ for 1 h, washed with pre-warmed tissue culture medium 3 times with gentle shaking (100 rpm) for 1 min each. The CIE were then either lysed in 0.1% Triton X-100 for 5 min, and the cell associated bacteria were recovered by plating lysates onto Luria agar, or processed for fluorescence microscopy for enumeration of bacteria attached to the cells.

### Fluorescence microscopy

To investigate the binding of FimH in the context of intestinal epithelial cells, 100 μl of 50 μg/ml biotinylated FimHLD or FimHLD:Q133K were incubated with CIE. After an hour of incubation at 37°C unbound FimHLD were washed off with tissue culture media, the cells were fixed with 2% paraformaldehyde for 30 min at room temperature (RT), washed twice with PBS, and blocked with 1% BSA-PBS for another 30 min at RT. Streptavidin coated Qdot 594 was then used at 1:100 dilution in 1% BSA-PBS to detect biotinylated FimHLD bound to the cell surface. For detection of ETEC adhesion, infected CIEs were incubated with anti-O78 antibody followed by fluorescent labeled secondary. Cell membranes and nuclei were stained as previously described (CellMask, red for membrane and DAPI for nuclei; Invitrogen) [61]. Images were acquired on a Zeiss LSM510 confocal microscope, and files were converted to TIFF image using ImageJ (v1.45). Signals were quantified using Volocity software (version 6.2; PerkinElmer, Inc.).

### Transmission immunoelectron microscopy

For localization of FimH on intestinal epithelium, ETEC infected CIE were fixed in 2% paraformaldehyde/0.02% glutaraldehyde (Polysciences Inc., Warrington, PA) in 100 mM PIPES/0.5 mM MgCl_2_, pH 7.2 for 1 h at 4°C. Samples were washed with PIPES buffer, blocked with 5% Fetal Bovine Serum /5% Normal Goat Serum for 20 min and subsequently incubated with rabbit anti-FimH antibody for 1 h, followed by secondary goat anti-rabbit antibody conjugated to 18 nm colloidal gold for 1 h. Samples were washed in buffer and postfixed in 1% osmium tetroxide (Polysciences Inc.) for 1 h. Samples were then rinsed extensively in dH_2_O prior to *en bloc* staining with 1% aqueous uranyl acetate (Ted Pella Inc., Redding, CA) for 1 h. Following several rinses in dH_2_O, samples were dehydrated in a graded series of ethanol and embedded in Eponate 12 resin (Ted Pella Inc.). Sections of 95 nm were cut with a Leica Ultracut UCT ultramicrotome (Leica Microsystems Inc., Bannockburn, IL), stained with uranyl acetate and lead citrate, and viewed on a JEOL 1200 EX transmission electron microscope (JEOL USA Inc., Peabody, MA) equipped with an AMT 8 megapixel digital camera (Advanced Microscopy Techniques, Woburn, MA).

### Growth and differentiation of human small intestinal epithelial cells

Enteroids were grown as previously described in detail [45] from purified intestinal cell lines, maintained at the Washington University Digestive Diseases Research Core Center BioSpecimens Core under a protocol approved by the Institutional Review Board. Briefly, cells were thawed and re-suspended in Matrigel (BD Biosciences, San Jose, CA, 15 μL/well in 24 well plates), and incubated at 37°C with a 1:1 mixture of L-WRN conditioned media (CM) and primary culture media (Advanced DEM/F12, Invitrogen) supplemented with 20% fetal bovine serum (FBS), 2 mM L-glutamine, 100 units/mL penicillin, 0.1 mg/mL streptomycin, 10 μM Y-27632 (ROCK inhibitor; Tocris Bioscience, R and D Systems, Minneapolis, MN), and 10 μM SB 431542 (transforming growth factor-ß type 1 receptor inhibitor; Tocris Bioscience, R and D Systems). For polarization, cells were added to Transwell filters (Corning) and incubated in differentiation media (1:20 mixture of L-WRN CM and primary culture media) lacking SB 431542. Cells were grown to confluence for 3 days in differentiation media before use.

### Histological analyses of eneteroid-derived human intestinal epithelial monolayers

For histological analysis (Day 3), enteroids grown on transwell were fixed in 3.7% paraformaldehyde (Electron Microscopy Sciences, Hatfield, PA) for 15 min, washed once with PBS and processed for paraffin embedding. Transverse sections (5 μm) were stained with hematoxylin and eosin (visualized with a Zeiss Axioskop 2 MOT microscope fitted with a CRI Nuance FX multispectral imaging system, Cambridge Research and Instrumentation), or used for immunostaining. For immunostaining, sections were de-paraffinized and hydrated, boiled in Unmasking Solution (Vector Laboratories, Burlingame, CA) for 25 min, rinsed in PBS, blocked in 1% bovine serum albumin/0.1% Triton-X100 for 30 min and incubated with primary antibody at 4°C overnight. Primary antibodies included rabbit anti- ChgA (1:100, Abcam, Cambridge, MA), rabbit anti-Muc2 (1:100, Santa Cruz Biotechnology, Inc., Dallas, Texas), mouse monoclonal anti-Villin 1 (1:100; Santa Cruz Biotechnology, Inc.). Slides were rinsed 3 times with PBS and incubated for 60 min at RT with species specific secondary antibodies (1:200; Invitrogen) conjugated to AlexaFluor488 or AlexaFluor546. For detection of bacteria, enteroids (grown for 3 days) were infected for 1 h, washed 3 times with pre-warmed media, fixed in 3.7% paraformaldehyde and either processed for fluorescent microscopy or embedded in paraffin. For the detection of tight junction formation, antibody against zonula occludens-1 (anti-ZO-1, mouse monoclonal, Invitrogen) was used at 1:100 dilutions followed by anti-mouse secondary antibodies conjugated to AlexaFluor488 (1:200, Invitrogen). Paraffin embedded sections were deparaffinized and processed for immunostaining with anti-O78 antibody (1:200) followed by secondary antibodies (1:200; Invitrogen) conjugated to AlexaFluor 546 for the detection of cell associated bacteria. UEA-1 lectin conjugated to FITC (1:100, Sigma) was applied during secondary antibody incubation. Slides were washed 3 times in PBS and stained with DAPI (Molecular Probe) to visualize nuclei and cellmask red (1:2000, Invitrogen) to visualize plasma membrane and mounted with ProlongGold antifade reagent (Molecular Probes) for confocal microscopy (Nikon ECLIPSE Ti confocal microscope equipped with NIS-Elements imaging software). To examine ETEC association with microvilli of polarized primary human small intestinal epithelial cells, infected monolayers were processed for transmission electron microscopy following protocol mentioned above.

### Toxin secretion assay

LT toxin secretion by different strains was measured using previously established GM1-ELISA method [81]. Briefly, ELISA plates (Costar, Corning, NY) were coated with 100 μl/well of 1 μg/ml monosialoganglioside GM1 (Sigma, G-7641), and incubated overnight at RT. After blocking with 2% BSA in PBS-Tween, 100 μl of clarified culture supernatant from overnight Luria broth cultures of bacteria was added to wells in triplicate and incubated for 1 h at 37°C. The wells were then washed and incubated with 100 μl of a 1:5000 dilution of rabbit anti-LTB polyclonal antisera for 1 h. Plates were again washed and then incubated with 100 μl of a 1:5000 dilution of anti-rabbit secondary conjugated with HRP for another hour. Finally plates were developed with 100 μL per well of TMB-H_2_O_2_ (KPL) substrates and read immediately at 630nm for kinetic measurement (Eon, BioTek instruments, VT, USA).

### Cyclic nucleotide assays

Caco-2 cell monolayers grown in 96-well plates were infected with WT or mutant strains and used to determine alterations in cAMP. After 2 h of infection wells were washed with pre-warmed tissue culture media, incubated for an additional 2.5 h, cells lysed in sample buffer and intracellular cAMP concentrations were then determined by ELISA (cAMP Direct EIA, Arbor Assays, MI, USA). Similarly, T-84 cell monolayers grown in 96-well plates were used to determine cGMP concentrations following infection. 1.5 h after infection, wells were washed 3 times with pre-warmed tissue culture media, incubated for an additional 3 h, lysed in sample buffer, and intracellular cGMP determined (cGMP Direct EIA, Arbor Assays, MI, USA).

### Rabbit ileal loop assays

The rabbit ileal loop (RIL) assay was performed as previously described [49, 82]. Briefly, bacteria were grown under static conditions in Luria broth at 37°C, then washed with PBS, and diluted yield to a target inoculum of ~1X10^6^ CFU/ml/loop. Adult albino rabbits (New Zealand strain) weighing 1.5–2.0 kg were fasted for 24 h and allowed only water. Following induction of anesthesia with intravenous sodium pentobarbital (0.5 mL/kg body mass), the abdominal cavity was entered, the intestine exposed and the ileum localized. Here, loops of 5 cm in length were created with surgical ligatures, with 2 cm intervals between each loop to yield 5–6 loops per rabbit. Loops were then inoculated with either wild type H10407 as a positive control or mutant strains. Animals were euthanized ~18 h post infection with sodium pentobarbital, the intestine exposed and the ileocaecal region was then removed. The volume of fluid accumulation determined per unit length of gut within each segment, after which tissue specimens from the corresponding segment were collected, fixed in formalin (10%), paraffin-embedded and subsequently processed for examination of villous associated bacteria following protocol mentioned above.

### Ethics statement

Rabbit ileal loop protocol conducted at icddr,b was reviewed and approved by the institution’s Animal Experimentation Ethics Committee (AEEC). Experimental procedures were carried out by veterinarians at the animal facility in accordance with relevant guidelines. The rules and guidelines followed at icddr,b for animal care and use adhere to the recommendations, with some modifications, stated in the Guide for the Care and Use of Laboratory Animals of the National Institutes of Health (NIH). These guidelines and the subsequent modifications were approved by the icddr,b Board of Trustees. All work involving cell lines derived from human biopsy specimens with the Washington University Digestive Diseases Research Core Center BioSpecimens Core was performed under a protocol (201406083) approved by the Institutional Review Board at Washington University School Medicine.

## Supporting information

S1 Fig Type 1 pili mediated ETEC adhesion to epithelial cells. **a.** Adhesion by wild type (wt) bacteria or the *fimH*::Q133K mutant. The percentage of cell associated bacteria represents the proportion of bacteria associated with the epithelial cells 1 h post infection relative to the inoculum. **b.** *Adhesion assays of* wt, *fimA* mutant and *fimA* mutant complemented *in trans* with pFimA, or the vector control plasmid. Yeast agglutination phenotypes for the wt and mutant bacteria are shown below each column of data in the graphs. For data in parts **a** and **b** bars represent the mean. Error bar, SEM (n = 5). P values were calculated by nonparametric Mann-Whitney test. ** represents p<0.01; *p<0.05.
(TIF)

S2 Fig Type 1 pili act in concert with CFA/I fimbriae for optimal adhesion. *In vitro* adhesion assay to Caco-2 cells infected with either WT H10407 or different mutants, including *fimH* and *cfaE* single mutants or *fimH-cfaE* double mutants. The percentage of cell associated bacteria represents the proportion of bacteria associated with Caco-2 cells at the end of 1 h relative to the inoculum. Different color dots represent data from different experiments, horizontal dashed lines represent geometric mean values. P values were calculated by nonparametric Mann-Whitney test. *** indicates p<0.0001.
(TIF)

S1 Table Strains used in this study. Km^R^ = kanamycin resistance cassette; Cm^R^ = chloramphenicol resistance cassette (chlormaphenicol acetyltransferase; CAT).
(DOCX)

S2 Table Primers used in this study. p1 and p2 regions are as originally defined by Datsenko, *et al* [76]. cat = chloramphenicol acetyl transferase.
(DOCX)

S3 Table Plasmids used in this study. Amp^R^ = ampicillin resistance cassette; Km^R^ = kanamycin resistance cassette.
(DOCX)
