# Supplementary figures and images for: Highly conserved type 1 pili promote enterotoxigenic E. coli pathogen-host interactions
Source: PLoS Negl Trop Dis. 2017 May 22;11(5):e0005586. doi: 10.1371/journal.pntd.0005586 (PMC5456409; doi:10.1371/journal.pntd.0005586)

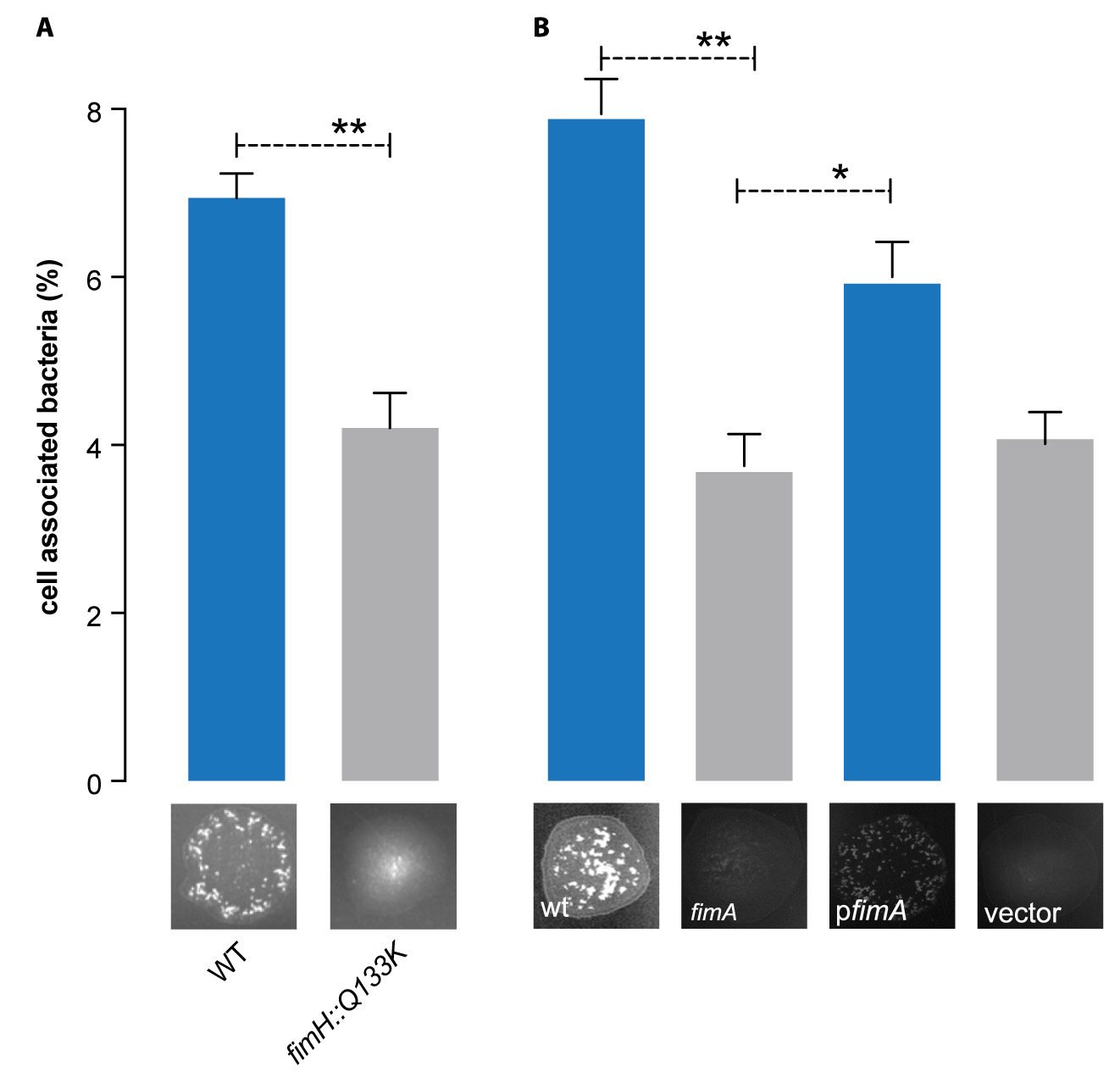

Supplement: S1 Fig — a. Adhesion by wild type (wt) bacteria or the fimH::Q133K mutant. The percentage of cell associated bacteria represents the proportion of bacteria associated with the epithelial cells 1 h post infection relative to the inoculum. b. Adhesion assays of wt, fimA mutant and fimA mutant complemented in trans with pFimA, or the vector control plasmid. Yeast agglutination phenotypes for the wt and mutant bacteria are shown below each column of data in the graphs. For data in parts a and b bars represent the mean. Error bar, SEM (n = 5). P values were calculated by nonparametric Mann-Whitney test. ** represents p<0.01; *p<0.05. (TIF) [file pntd.0005586.s001.tif]

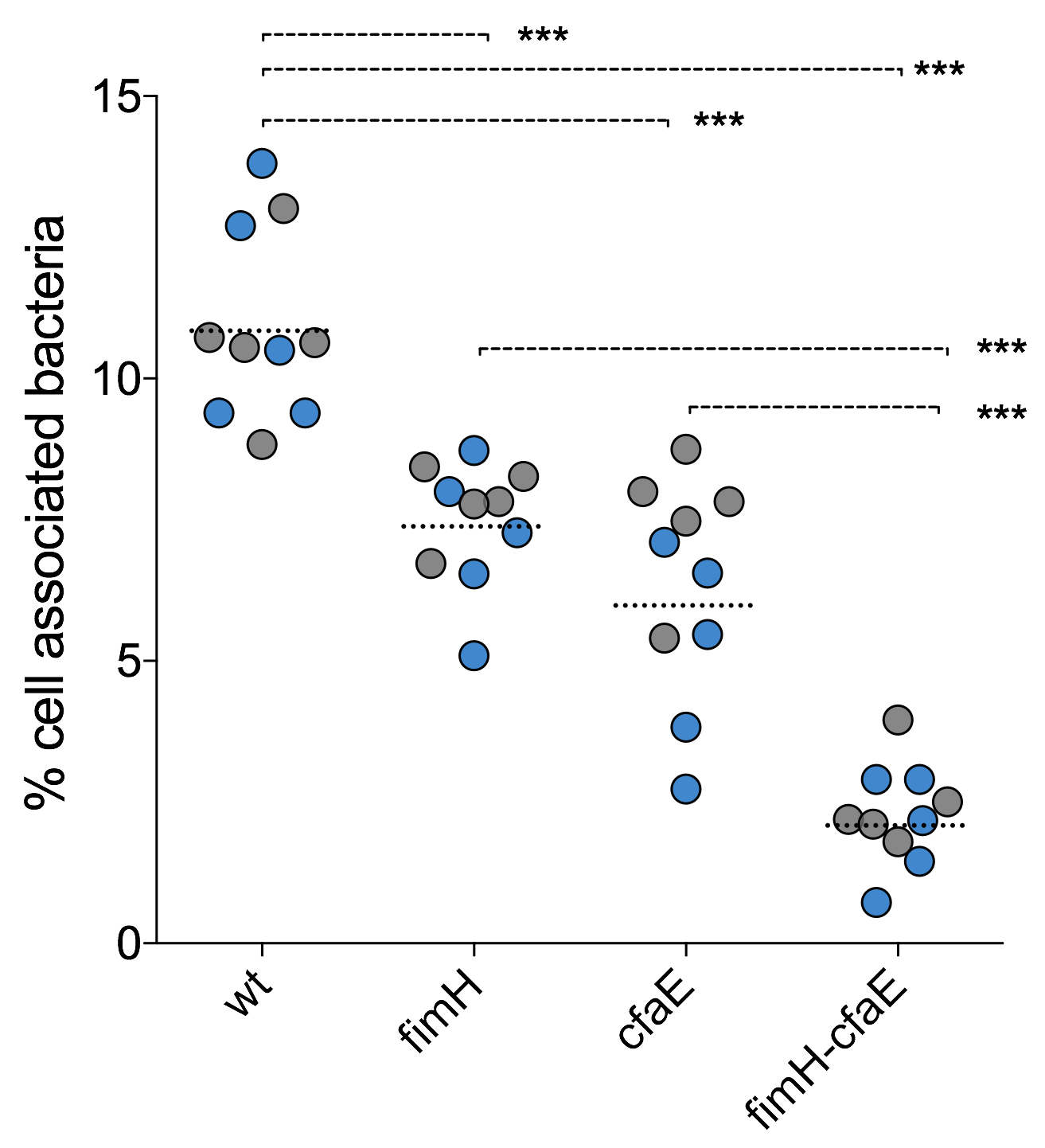

Supplement: S2 Fig — In vitro adhesion assay to Caco-2 cells infected with either WT H10407 or different mutants, including fimH and cfaE single mutants or fimH-cfaE double mutants. The percentage of cell associated bacteria represents the proportion of bacteria associated with Caco-2 cells at the end of 1 h relative to the inoculum. Different color dots represent data from different experiments, horizontal dashed lines represent geometric mean values. P values were calculated by nonparametric Mann-Whitney test. *** indicates p<0.0001. (TIF) [file pntd.0005586.s002.tif]
