# Supplementary material for: Highly conserved type 1 pili promote enterotoxigenic E. coli pathogen-host interactions
Source: PLoS Negl Trop Dis. 2017 May 22;11(5):e0005586. doi: 10.1371/journal.pntd.0005586 (PMC5456409; doi:10.1371/journal.pntd.0005586)
Supplement: S1 Table — KmR = kanamycin resistance cassette; CmR = chloramphenicol resistance cassette (chlormaphenicol acetyltransferase; CAT). (DOCX) [file pntd.0005586.s003.docx]

**S1 Table. Strains used in this study**

| **strain**  **designation** | Genotype | Description | Reference |
| --- | --- | --- | --- |
| H10407 | wild type | ETEC serotype 078:H11, LT^+^LST^+^ | [78] |
| jf570 | *eltAB* | H10407 derivative with deletion of genes encoding heat-labile toxin (LT) | [[47](#_ENREF_47)] |
| jf2944 | *fimH::Km^R^* | *fimH*; *fimH* gene is replaced with kanamycin resistant cassette | This study |
| jf4622 | *fimH::Km^R^* (p*fimH*) | *fimH* mutants complemented with p*fimH* | This study |
| jf4624 | *fimH::Km^R^* (p*fimH:Q133K*) | *fimH* mutants complemented with mutant allele of *fimH* on p*fimH:Q133K*; kanamycin and ampicillin resistant | This study |
| jf2003 | *fimA::Km^R^* | *fimA* gene replaced by kanamycin resistance cassette | This study |
| jf2051 | *fimA:: Km^R^* (p*fimA*) | *fimA* mutants complemented with p*fimA*; kanamycin and ampicillin resistant | This study |
| jf2379 | *fimA:: Km^R^* (pTrc99A) | *fimA* mutant complemented with control vector (pTrc99A) plasmid, kanamycin and ampicillin resistant | This study |
| jf1862 | *cfaE::Km^R^* | *cfaE* gene replaced with kanamycin resistant cassette | [[40](#_ENREF_40)] |
| jf2945 | *fimH:: Km^R^*, *cfaE::cm* | *fimH-cfaE* double mutants; *fimH* gene is replaced with chloramphenicol resistant cassette and *cfaE* gene is replaced with kanamycin resistant cassette | This study |
| jf2847 | *estH::Cm^R^, estP:: Km^R^* | H10407 derivative with deletion of ST toxin encoding genes; *estH* gene is replaced with chloramphenicol resistant cassette and *estP* gene is replaced with kanamycin resistant cassette | This study |
| jf4615 | BL21DE3 pLys (p*fimHLD-his*) | fimHLD expression strain; Ampicillin resistant | This study |
| jf4617 | BL21DE3 pLys (p*fimHLD:Q133K-his*) | mutant fimHLD expression strain, Ampicillin resistant | This study |
| jf4638 | *fimH*:Q133K | H10407-Q133K; H10407 derived strain with a point mutation introduced in the mannose binding pocket of *fimH* on the chromosome | This study |
