# Supplementary material for: Highly conserved type 1 pili promote enterotoxigenic E. coli pathogen-host interactions
Source: PLoS Negl Trop Dis. 2017 May 22;11(5):e0005586. doi: 10.1371/journal.pntd.0005586 (PMC5456409; doi:10.1371/journal.pntd.0005586)
Supplement: S2 Table — p1 and p2 regions are as originally defined by Datsenko, et al [76]. cat = chloramphenicol acetyl transferase. (DOCX) [file pntd.0005586.s004.docx]

**S2 Table. Primers used in this study**

| Primers | Sequence | | Description |
| --- | --- | --- | --- |
| jf101413.7 | CCATTCAGGCAGTGATTAGCATCACCTATACCTACAGCTGAACCCGAAGAGATGATTGTAGTGTAGGCTGGAGCTGCTTC | | forward *fimH* deletion primer; 60 nucleotides homology tail immediately upstream from *fimH*. p1 region of pKD4 |
| jf101413.8 | AAGGGCTAACGTGCAGGTTTTGTAGGTCTGATAGCGTAGCGCCTCAGGTACCAGCATTAGCATATGAATATCCTCCTTA | | reverse *fimH* deletion primer; 60 nucleotide homology tail immediately downstream from *fimH*. p2 region of pKD4 |
| jf101413.9 | TGGCAACACATTGAATACTGG | | forward *fimH* mutant test primer |
| jf101413.10 | TGCCAGATGCGACGCTGACGC | | reverse *fimH* mutant test primer |
| jf062116.1 | GAATTTGTAAAGAACCCACGTGTGCAGGATTTGCTGGCAAAGAATGATAAAGGATAAACGGTTTAAACGATATCGGATCCA | | forward *cfaE* deletion primer; 60 nucleotide homology tail immediately upstream from *cfaE*. nucleotide sequence of *cat* gene |
| jf062116.2 | TTAACAAACAGATTACCTATTTACAATATTGGCGCGCAATAGCGCCAATATTGTTGTTATACTAGTATTACCCTGTTATCC | | reverse *cfaE* deletion primer; 60 nucleotide homology tail immediately downstream from *cfaE*. nucleotide sequence of *cat* gene |
| jf042314.1 | AGGAGATATACCATGATGAAACGAGTTATTACCCTGT | forward In-Fusion primer; nucleotides upstream of *NcoI* cloning site on pETDUET1.  22 nucleotides of *fimH* from the start codon site | |
| jf042314.2 | ATGCGGCCGCAAGCT**TTAGTGGTGATGATGGTGATG**GCCGCCAGTGGGCACCAC | | reverse In-Fusion primer; nucleotides downstream of *HindIII* cloning site of pETDUET1.  **nucleotides for 6xhistidine tag with stop codon**. last 18 nucleotides of *fimH* without stop codon |
| jf120814.1 | ATATCATATGAAGCTATGAAACGAGTTATTACCCTGT | | forward In-fusion primer; nucleotides upstream of *HindIII* cloning site of pFLAG-CTC.  22 nucleotides of *fimH* from the start codon site |
| jf120814.2 | TGTAGTCGACAGATCTTATTGATAAACAAAAGTCACG | | reverse, In-Fusion primer; nucleotides downstream of *HindIII* cloning site of pFLAG-CTC. last 22 nucleotides of *fimH* with stop codon |
| jf031814.1 | CTCATTAATTGCCGTGCTTATTTTGCGAAAGACCAACAACTATA | | forward mutagenesis primer for Q133K allele; point mutation is underlined |
| jf031814.2 | TATAGTTGTTGGTCTTTCGCAAAATAAGCACGGCAATTAATGAG | | reverse mutagenesis primer for Q133K allele; point mutation is underlined |
