# Supplementary material for: Highly conserved type 1 pili promote enterotoxigenic E. coli pathogen-host interactions
Source: PLoS Negl Trop Dis. 2017 May 22;11(5):e0005586. doi: 10.1371/journal.pntd.0005586 (PMC5456409; doi:10.1371/journal.pntd.0005586)
Supplement: S3 Table — AmpR = ampicillin resistance cassette; KmR = kanamycin resistance cassette. (DOCX) [file pntd.0005586.s005.docx]

**S3 Table. Plasmids used in this study**

| Plasmid | Description | Reference |
| --- | --- | --- |
| p*fimH* | Complementation plasmid; *fimH* gene cloned into pFLAG-CTC Amp^R^ | This study |
| p*fimA* | *fimA* gene cloned into of EcoR1/BamHI sites of pTrc99A | this study |
| p*fimH:Q133K* | Complementation plasmid; *fimH:Q133K* mutant allele cloned into pFLAG-CTC, Amp^R^ | This study |
| pETDUET1-fimHLD-his | Expression plasmid for purification of FimH lectin domain (FimHLD),Amp^R^ | This study |
| pETDUET1-fimHLD:Q133K-his | Expression plasmid for purification of mutated FimH lectin domain (FimHLD:Q133K), Amp^R^ | This study |
| pFLAG-CTC | expression plasmid vector, Amp^r^ | Sigma |
| pTrc99A | pBR322-derived expression plasmid vector; Amp^r^ | [77] |
| pETDUET-1 | expression plasmid, Amp^R^ | Novagen |
| pKD46 | Helper plasmid for λ-Red mediated recombination, Amp^R^ | [76] |
| pKD4 | Template plasmid for kanamycin resistance cassette amplification, Km^R^ | [76] |
